# Supplementary material for: Three-Year Longitudinal Study: Prevalence of Salmonella Enterica in Chicken Meat is Higher in Supermarkets than Wet Markets from Mexico
Source: Foods. 2020 Mar 2;9(3):264. doi: 10.3390/foods9030264 (PMC7143798; doi:10.3390/foods9030264)
Supplement: Supplementary file 1 [file foods-09-00264-s001.pdf]

Supplementary tables and figures

Results of analyses using equal number of samples (n = 240) for year 2016, 2017, and 2019.

Table 1S. *Salmonella* Prevalence over time.

	**No. of samples**	**Prevalence (%)**	**95% CI**
**Overall**			
2016 - 2018	720	20.7	17.9 - 23.8
**Annual**			
2016	240	13.3	9.6 - 18.2
2017	240	21.7	16.7 - 27.5
2018	240	27.1	21.7 - 33.3
**Seasonal**			
Spring	180	17.8	12.9 – 24.0
Summer	180	20.0	14.8 - 26.4
Autumn	180	23.3	17.8 – 30.0
Winter	180	21.7	16.3 - 28.3
**Monthly**			
January	60	20.0	11.8 - 31.8
February	60	20.0	11.8 - 31.8
March	60	15.0	8.1 - 26.1
April	60	21.7	13.1 - 33.6
May	60	16.7	9.3 - 28.0
June	60	13.3	6.9 - 24.1
July	60	25.0	15.8 - 37.2
August	60	21.7	13.1 - 33.6
September	60	20.0	11.8 - 31.8
October	60	20.0	11.8 - 31.8
November	60	30.0	19.9 - 42.5
December	60	25.0	15.8 - 37.2

Table 2S. *Seasonal* effect on Salmonella prevalence per year.

Season	2016	2017	2018
Spring	9/60 (15)^{ab}	10/60 (16.7)^{a}	13/60 (21.7)^{a}
Summer	5/60 (8.3)^{b}	15/60 (25.0)^{a}	16/60 (26.7)^{a}
Autumn	5/60 (8.3)^{b}	18/60 (30.0)^{a}	19/60 (31.7)^{a}
Winter	13/60 (21.7)^{a}	9/60 (15.0)^{a}	17/60 (28.3)^{a}

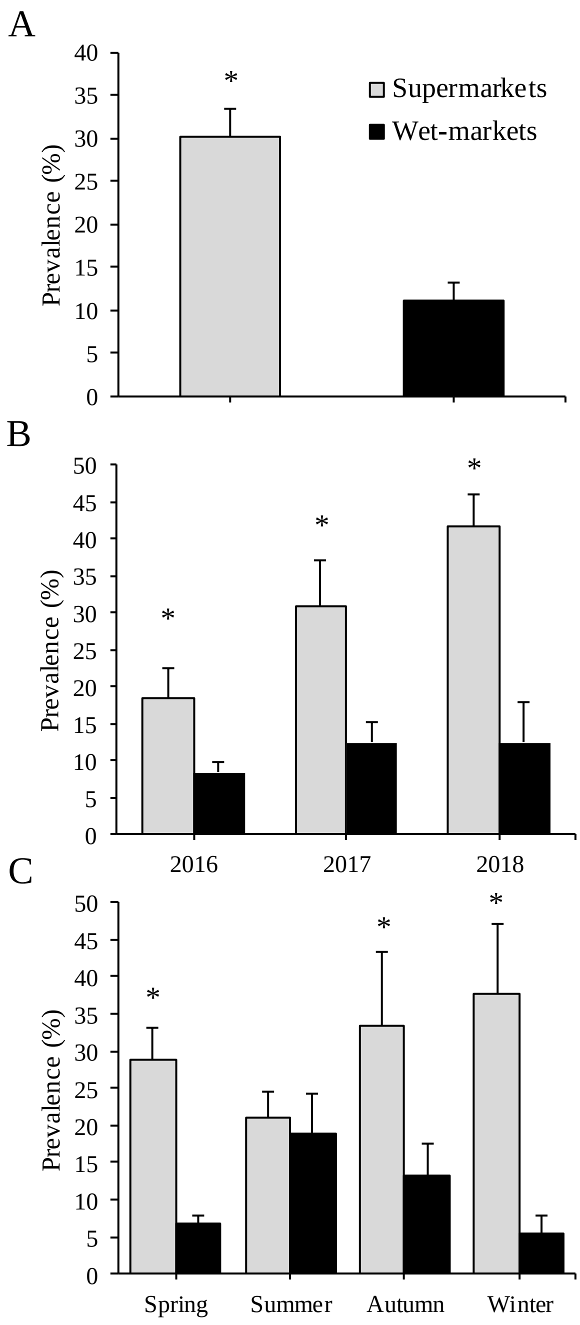

Figure 1S. Influence by market type (A), over time (B) and season effect (C) on *Salmonella* prevalence in chicken meat.

Table 3S. Odds ratio analysis of the *Salmonella* prevalence over time.

	**Odds ratio**	**95 % confidence interval**	***P* value**
**Overall**			
2016 - 2018	2.4	1.5 - 3.8	***P < 0.001***
**Months**			
January	3.8	0.9 - 16.0	***P = 0.0635***
February	16.8	2.0 - 140.9	***P = 0.0094***
March	4.3	0.8 - 22.5	*P = 0.0880*
April	4.5	1.1 - 18.5	***P = 0.0371***
May	12.4	1.5 - 105.7	***P = 0.0211***
June	3.5	0.6 - 19.0	***P = 0.1464***
July	0.8	0.2 - 2.6	*P = 0.7657*
August	0.8	0.2 - 2.8	*P = 0.7542*
September	0.7	0.2 - 2.4	*P = 0.5202*
October	7.0	1.4 - 35.5	***P = 0.0188***
November	9.0	2.2 - 36.2	***P = 0.0020***
December	25.4	3.1 - 211.1	***P = 0.0028***
**Season**			
Spring	5.7	2.2 - 14.6	***P = 0.0003***
Summer	1.2	0.6 - 2.4	*P = 0.7095*
Autumn	3.25	1.5 - 6.9	***P = 0.0020***
Winter	10.3	3.8 - 28.0	***P < 0.0001***
**Year**			
2016	2.5	1.1 - 5.5	***P = 0.0259***
2017	3.1	1.6 - 6.1	***P = 0.0008***
2018	5.0	2.6 - 9.6	***P < 0.0001***

*Reference: Wet-market (odds ratio = 1).

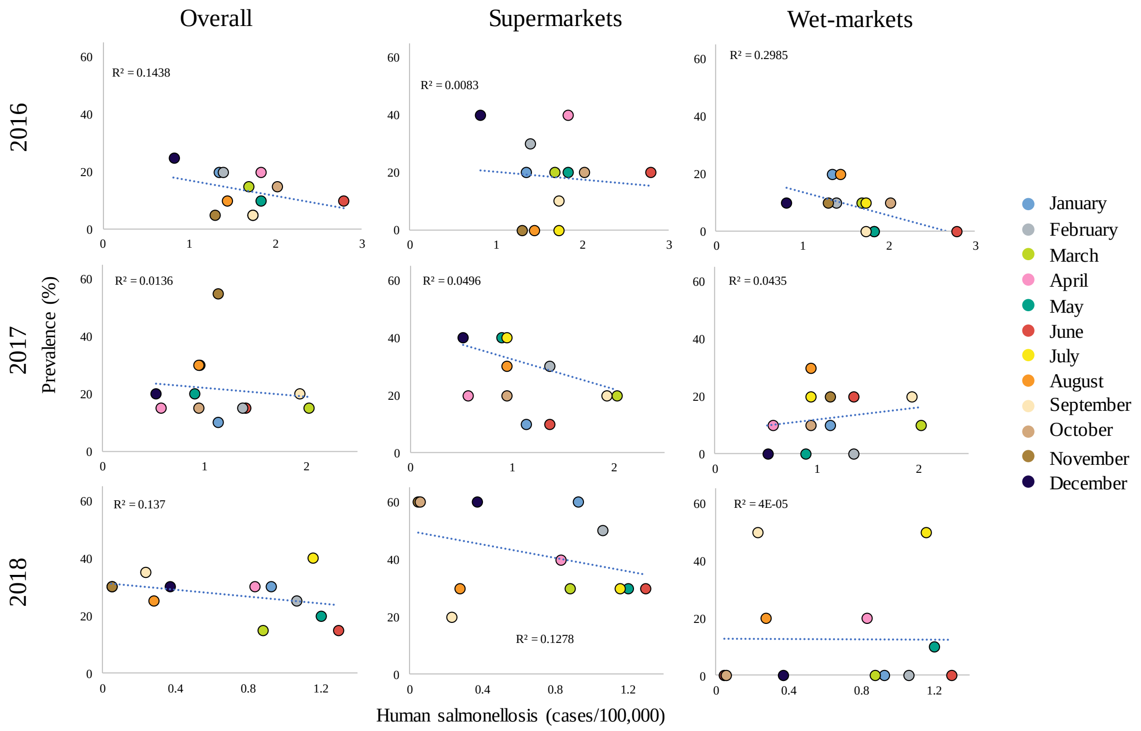

Figure 2S. Correlation between *Salmonella* prevalence in retail chicken meat and human salmonellosis cases.
